# Functional Tissue Analysis Reveals Successful Cryopreservation of Human Osteoarthritic Synovium

**DOI:** 10.1371/journal.pone.0167076

**Published:** 2016-11-21

**Authors:** Mathijs G. A. Broeren, Marieke de Vries, Miranda B. Bennink, Peter L. E. M. van Lent, Peter M. van der Kraan, Marije I. Koenders, Rogier M. Thurlings, Fons A. J. van de Loo

**Affiliations:** Experimental Rheumatology, Department of Rheumatology, Radboud Institute for Molecular Life Sciences, Radboud University Medical Center, Nijmegen, the Netherlands; EFS, FRANCE

## Abstract

Osteoarthritis (OA) is a degenerative joint disease affecting cartilage and is the most common form of arthritis worldwide. One third of OA patients have severe synovitis and less than 10% have no evidence of synovitis. Moreover, synovitis is predictive for more severe disease progression. This offers a target for therapy but more research on the pathophysiological processes in the synovial tissue of these patients is needed. Functional studies performed with synovial tissue will be more approachable when this material, that becomes available by joint replacement surgery, can be stored for later use. We set out to determine the consequences of slow-freezing of human OA synovial tissue. Therefore, we validated a method that can be applied in every routine laboratory and performed a comparative study of five cryoprotective agent (CPA) solutions. To determine possible deleterious cryopreservation-thaw effects on viability, the synovial tissue architecture, metabolic activity, RNA quality, expression of cryopreservation associated stress genes, and expression of OA characteristic disease genes was studied. Furthermore, the biological activity of the cryopreserved tissue was determined by measuring cytokine secretion induced by the TLR ligands lipopolysaccharides and Pam3Cys. Compared to non frozen synovium, no difference in cell and tissue morphology could be identified in the conditions using the CS10, standard and CryoSFM CPA solution for cryopreservation. However, we observed significantly lower preservation of tissue morphology with the Biofreeze and CS2 media. The other viability assays showed trends in the same direction but were not sensitive enough to detect significant differences between conditions. In all assays tested a clearly lower viability was detected in the condition in which synovium was frozen without CPA solution. This detailed analysis showed that OA synovial tissue explants can be cryopreserved while maintaining the morphology, viability and phenotypical response after thawing, offering enhanced opportunities for human *in vitro* studies.

## Introduction

Osteoarthritis is a common joint disease characterized by degenerative alterations in the articular cartilage. However, it is becoming more evident that OA is not just a wear and tear disease of the cartilage but involves multiple joint tissues including the synovium [1,2]. The synovium is the tissue lining the joint capsule and in OA often shows signs of inflammation, for example by macrophages infiltrating the synovium. These macrophages produce pro-inflammatory cytokines like IL1β and TNFα, activating the fibroblast-like synoviocytes to release additional cytokines (IL6, IL8) and matrix-degrading enzymes [3]. These cytokines and enzymes damage the articular cartilage, resulting in the release of damage-associated molecular patterns (DAMPS) that have pro-inflammatory properties as well, creating a perpetuating loop causing low grade chronic inflammation [3,4]. This inflammation is suggested to contribute to the disease phenotype and disease progression [1,2,5]. Studying the fundamental pathological processes in the OA synovium would help in finding a treatment for this disease.

Of importance for such studies is the availability of synovial tissue. Patient material becomes available as remnant material after joint replacement surgery, or via synovial biopsy. Functional studies on synovial tissue are often limited to a small number of patients as material needs to be used immediately when becoming available. Thus, timing is an important issue, hampering optimal planning and synchronization of an experiment. A solution would be to cryopreserve the valuable synovial tissue in such a way that the thawed synovial tissue performs identical to freshly obtained material. To our knowledge there are no studies describing cryopreservation of human OA synovial tissue in which the disease characteristic cell functions and interactions are maintained.

Although cryopreservation of cell lines is widely and successfully applied in almost every cell culture lab, the cryopreservation of primary cells and especially complex tissues is still challenging [6–11], as no regeneration takes place. Furthermore, a tissue is composed of a mixture of cell types, all with their specific optimal cryopreservation requirements [8,10]. Size is another obstacle, as this influences CPA solution penetration and leads to differences in exposure to CPAs [12]. Globally, two cryopreservation methods exist, slow freezing and vitrification. Vitrification has theoretical advantages over slow freezing once the optimal conditions are determined. However, the high CPA concentration needed to vitrify the material is a major drawback, due to their toxicity [10]. Slow freezing uses low concentrations of CPA, reducing CPA toxicity. Another advantage of slow freezing is that the procedure to get the tissue ‘freezer-ready’ is fast and easy to apply in every routine laboratory without needing advanced equipment.

We have compared four commercially available and one homemade CPA solution for their suitability to cryopreserve human OA synovial tissue. These CPA solutions are developed for application in a slow freezing protocol. To validate our method we studied the viability of the cells and the conservation of tissue-specific responses to inflammatory stimuli. Therefore, we studied the ability of the tissue to respond to the pathogen associated molecular pattern (PAMP) TLR ligands lipopolysaccharides (LPS) and Pam3Cys (P3C) by measuring cytokine secretion. Both DAMPs and PAMPs can activate macrophages and fibroblasts via the TLR-4 and TLR-2 pathway. TLR4 can be activated in similar fashion by the PAMP LPS and the OA related DAMP S100A8/9. TLR2 can be activated by the PAMP P3C and the OA related DAMP hyaluronic acid [13]. In previous studies PAMPs have been shown to upregulate several plasma proteins in the OA synovial fluid, similar to the DAMPs [13]. In this study we therefore included LPS and P3C to study the TLR-2/4 response.

This detailed analysis showed that OA synovial tissue explants can be cryopreserved while maintaining the morphology, viability and phenotypical response after thawing.

## Materials & Methods

### Synovial tissue

Synovial tissue was obtained as remnant material, after written informed consent, from OA patients undergoing joint replacement surgery at the Orthopedics department of the Radboud University Medical Center. Protocols were approved by the local committee on research involving human subjects (CMO region Arnhem-Nijmegen, the Netherlands) under NL-number 54055.091.15. Dissected synovial samples were immediately placed in Dulbecco’s modified Eagle’s medium (DMEM) (Gibco), supplemented with 1% penicillin–streptomycin at 4°C. To confirm synovial origin, characterized by the presence of a synovial lining, representative tissue samples were embedded in OCT and 6 μM cryosections were cut and subsequently stained by Hematoxilin and Eosin (H&E). Histology and general viability assays were performed with synovial tissue from three donors, gene expression analysis and cytokine secretion assays were performed with synovial tissue from four different donors. For microarray analysis synovial samples were obtained by surgery or via fine-needle arthroscopy from 10 OA patients and 7 controls.

### Cryopreservation procedure

Biopsies were punched out of the synovium using a disposable 3 mm biopsy punch (KAI medical) and collected in RPMI medium (Gibco) containing 10% FCS, pyruvate and pen/strep. Biopsies were transferred into a cryovial and 1 ml of CPA solution (4°) was added. Five CPA solutions were compared in this study: 1. CS2 (BioLife Solutions), 2. CS10 (BioLife Solutions), 3. Standard: 10% DMSO, 10% FCS in RPMI, 4. Cryo-SFM (PromoCell), 5.Biofreeze (Biochrom, Merck Millipore). The cryovial was incubated for 10 minutes on ice before being transferred to an isopropanol container (Nalgene, Mr. Frosty). The next day, the cryovials were relocated to liquid nitrogen where they were stored for one week. Thawing was performed by transferring the cryovial as quickly as possible from the liquid nitrogen into a 37°C water bath until all ice has disappeared. Subsequently, the tissue was washed 3 times 10 minutes in pre-warmed RPMI (37°C) before being cultured in RPMI (10% FCS, pyruvate and pen/strep) at 5% CO_2_, 37°.

### Histology and histological analysis

**Fig 1:**
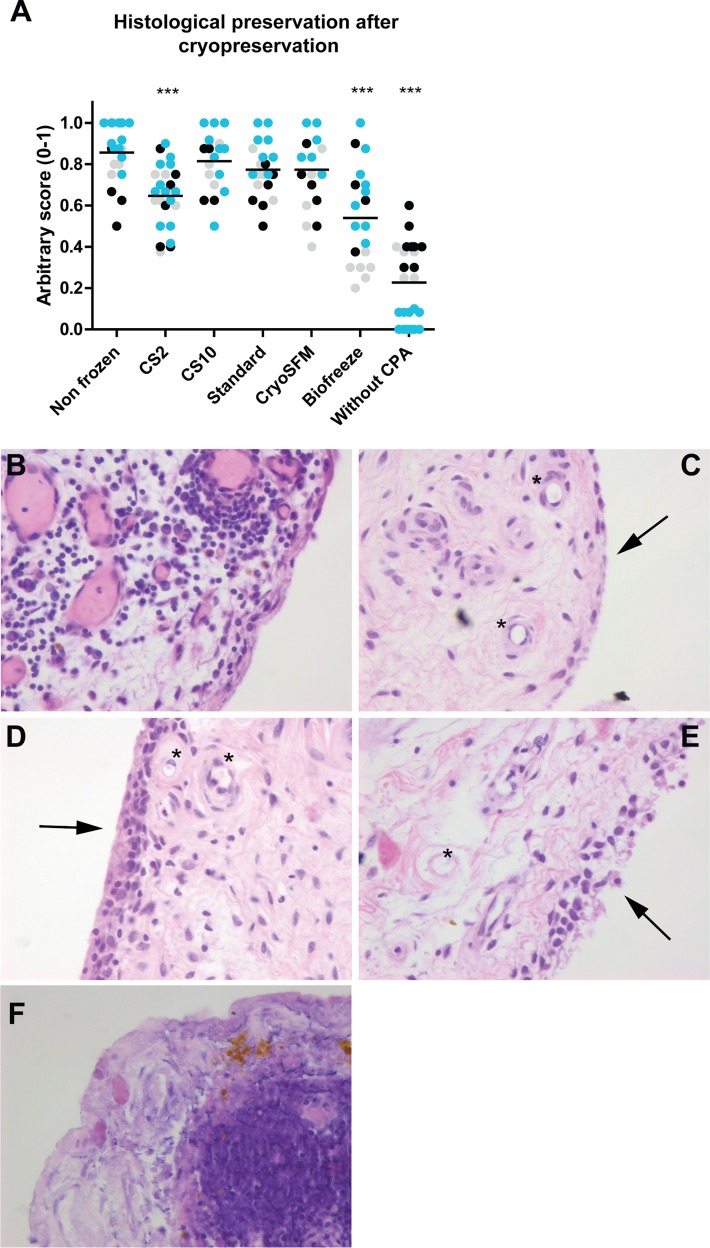
Histological analysis of cryopreserved synovial explants. (A) Histological preservation was determined by assessing global histological parameters using an arbitrary score. Mean score is displayed. A significantly lower score was determined for the CS2, Biofreeze and without CPA condition. Blue dots: patient 1, black dots: patient 2, grey dots: patient 3. For statistical analysis, one-way ANOVA was performed, comparing the non frozen control to the cryopreserved conditions. *** p<0.001. Displayed is the mean. (B) Non frozen highly cellular synovium from patient 1. (C) Synovium of low cellularity cryopreserved with CryoSFM of patient 2 displaying an intact and smooth intimal lining layer (arrow), intact cells and blood vessels (*). (D) Synovium from patient 3 cryopreserved with CryoSFM, displaying mainly intimal lining hyperplasia (arrow), the lining is smooth and intact, cells and blood vessels (*) are intact. (E) Synovium of patient 2 cryopreserved using the Biofreeze medium, showing a disrupted intimal lining layer (arrow), a high number of disrupted cells and disrupted blood vessels (*). (F) Synovium of patient 1 frozen without protecting CPA solution showing almost complete disruption of cells.

Non-frozen or thawed synovial biopsies from three patients were cultured for 24 hours before fixation in 4% formalin for 1 hour. For every condition, a minimum of 3 separate 3 mm biopsies was included. Fixation was followed by dehydration and embedding in paraffin. Sections were cut at 7 μm and stained by H&E. Histology of every biopsy was scored in duplo using an arbitrary scale (0–1) for global morphology, appearance of the intimal lining layer, cellular and nuclear morphology, morphology of infiltrating cells, blood vessel morphology and fat cell morphology. All parameters were given a score of 1, 0.5 or 0. A score of 1 indicated no observed change, a score of 0,5 was given when the morphology was clearly distinct from non-frozen and a score of 0 was given to complete structural loss and cell death of the tissue. The mean score of these parameters is displayed in Fig 1. Pictures were taken at 400x magnification.

### General viability assays

Non-frozen or thawed synovial biopsies were cultured overnight in a 96-wells plate in 100 μl RPMI (10% FCS, pyruvate and pen/strep) as described above. For the 2,3-bis-(2-methoxy-4-nitro-5-sulfophenyl)-2H-tetrazolium-5-carboxanilide (XTT) assay, 50 μl XTT reagent (Cell Proliferation Kit II, Roche) was added per well and the absorbance was measured at 450 and 655 nm after 2, 4 and 6 hours using a BioRad iMark microplate reader. Values were corrected for the reference wavelength and background signal. For the adenosine triphosphate (ATP) assay, 100 μl lysis buffer (Promega, Cell-Titer Glo) was added to each well and vigorously shaken for 5 minutes before incubation for 25 minutes at room temperature. Luminescence was determined using the BMG Labtech CLARIOstar microplate reader. Values were corrected for the background signal and depicted as relative light units (RLU).

### RNA isolation

Non-frozen and thawed synovial biopsies were cultured for 24 hours and subsequently snap-frozen in liquid nitrogen. Total RNA was isolated using the RNeasy fibrous tissue kit (Qiagen, Venlo, The Netherlands). Frozen synovial samples were transferred to MagNA Lyser Green Beads tubes (Roche) containing RLT lysis buffer. The tissue was disrupted in the MagNA lyser for 3 x 20 sec at 6000 rpm with one minute cooling between cycles. The remainder of the procedure was performed according to manufacturer instructions with on column DNA digestion.

### RNA integrity assessments

The RNA integrity number (RIN) was determined directly after RNA isolation with the Agilent RNA 6000 Nano kit using the protocol provided by the manufacturer. The Nano chip was run on the Agilent 2100 Bioanalyzer using the 2100 Expert Software.

### Microarray analysis

Microarray analysis was performed as described earlier [14]. Briefly, total RNA was isolated from the synovial samples, using the RNeasy kit for fibrous tissues (Qiagen, Venlo, The Netherlands). Total RNA (100 ng) was hybridized to a U133Plus 2.0 oligonucleotide array (Affymetrix, Santa Clara, CA). The arrays were scanned with a GeneChip scanner (Affymetrix) and analyzed with GeneChip operating software version 1.4 (Affymetrix). Array normalization and expression value calculations were performed with DNA-Chip Analyzer version 1.3 (www.dchip.org), using the invariant set normalization and the model-based method [15]. The OA synovial samples and control samples were deposited in the GEO database under accession number GSE82107.

### qPCR analysis

An unpublished systematic review of the literature was performed to find genes upregulated during cryopreservative stress. All Pubmed and Embase articles depicting cryopreservation and gene expression analysis of living human and animal cells and tissue were assessed and revealed six genes of which the expression is known to respond to cryopreservation conditions: two heat shock protein genes (*HSPA1A [16], HSP27 [17])* and four apoptosis-related genes (*CASP3 [18], BAX [19], CD95 [20] and MCL1[21]*). The expression of above mentioned genes was either increased or decreased (*HSPA1A*, *MCL1*) in comparison to non frozen cells or tissue. We determined their expression level after 24 hour culture.cDNA synthesis and qPCR analysis were performed as described previously[14]. Primer sequences are listed in S1 Table.

### Multiplex ELISA

Non-frozen and thawed synovial tissue was cultured for 24 hours in the presence or absence of LPS (100 ng/ml) and Pam3CSK4 (P3C) (1 μg/ml). Cytokine concentrations (IL1β, TNFα, IL-6 and IL-8) were determined in culture supernatants by Luminex multianalyte technology using the Bio-Plex 200 (Bio-Rad, Hercules, CA) and the Bio-Plex pro human cytokine kits (Bio-Rad) according to manufacturer instructions. To measure IL-6 and IL-8 levels, culture supernatants were diluted 200-fold.

### Statistics

**Fig 2:**
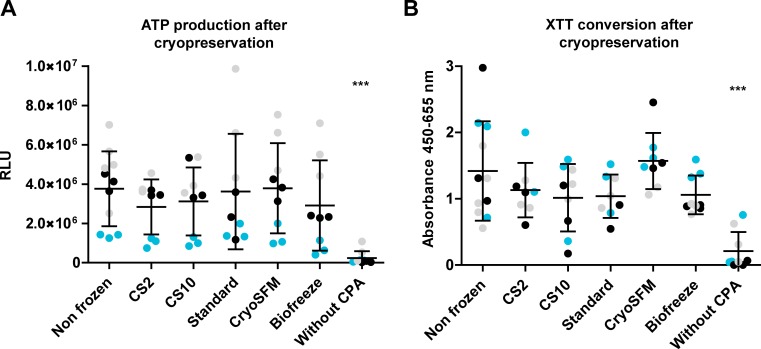
No change in metabolic activity in synovial explants after thawing. ATP and XTT levels were determined in triplo in OA synovial tissue of 3 patients. Patient 4: blue dots, patient 5: black dots, patient 1: grey dots. (A) Shown are the relative light units (RLU) (B) Shown is the percentage increase in absorbance over a 4 hour period, depicted as the mean +/- the SD. Statistical analysis was performed by one-way ANOVA, comparing the non-frozen control to the cryopreserved conditions. *** p<0.001.

**Fig 3:**
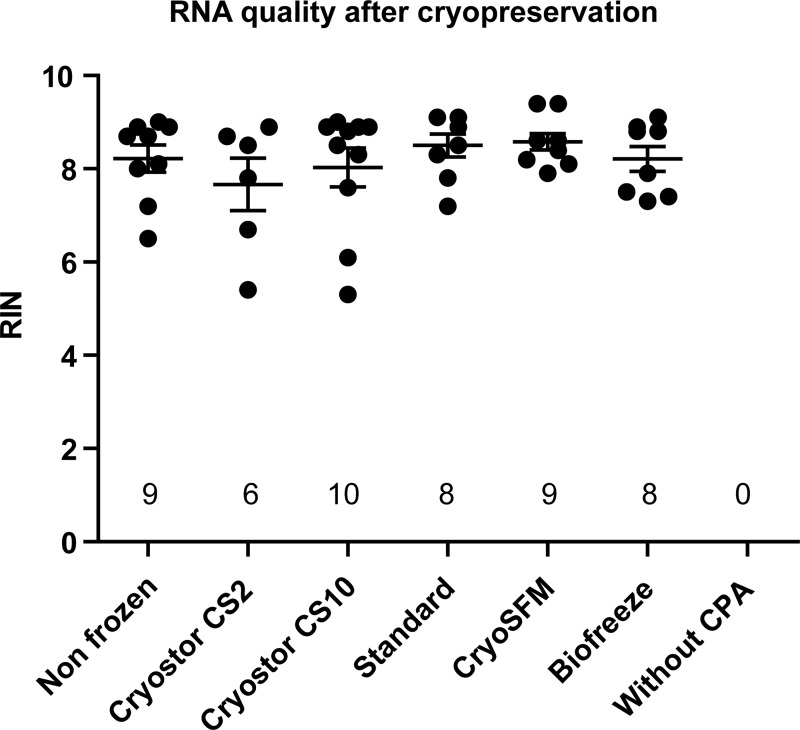
No effect of cryopreservation on RNA integrity in synovial explants. The RIN was determined in triplo of OA synovial tissue from four patients, 24 hours after thawing. Numbers indicate the number of biopsies of which the RIN could be successfully determined. Displayed is the mean +/- the SD. Statistical analysis was performed by one-way ANOVA, comparing the non-frozen control to the cryopreserved conditions.

**Fig 4:**
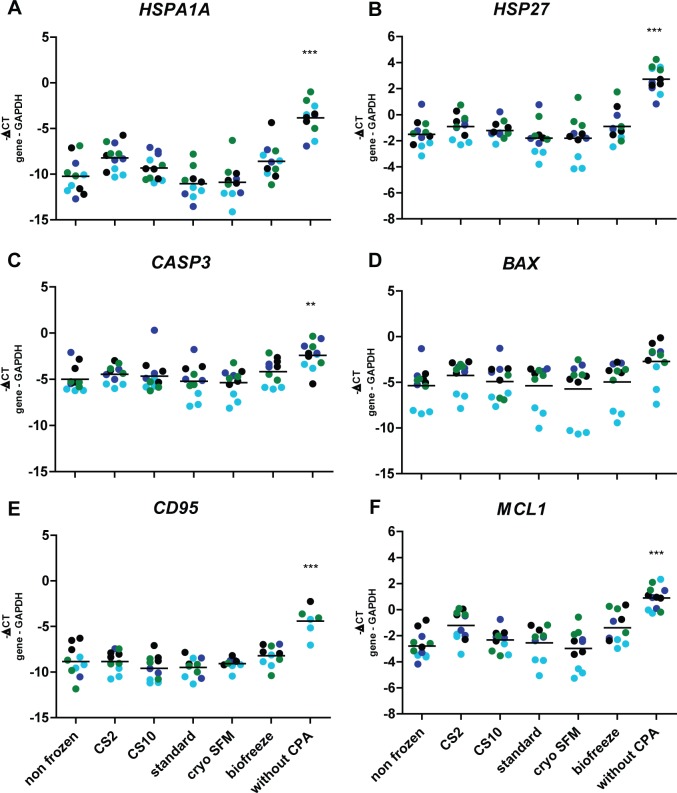
No increase in stress gene expression after cryopreservation. Gene expression of (A) *HSPA1a*, *(B) HSP27*, *(C) CASP3*, *(D) BAX*, *(E) CD95* and *(F) MCL1* was determined by qPCR analysis in four patients, 24 hours after thawing. Patient 6: green dots, patient 7: black dots, patient 8: dark blue dots, patient 4: light blue dots. Displayed is the mean. Statistical analysis was performed by one-way ANOVA, comparing the non-frozen control to the cryopreserved conditions. ** p<0.01; *** p<0.001.

**Fig 5:**
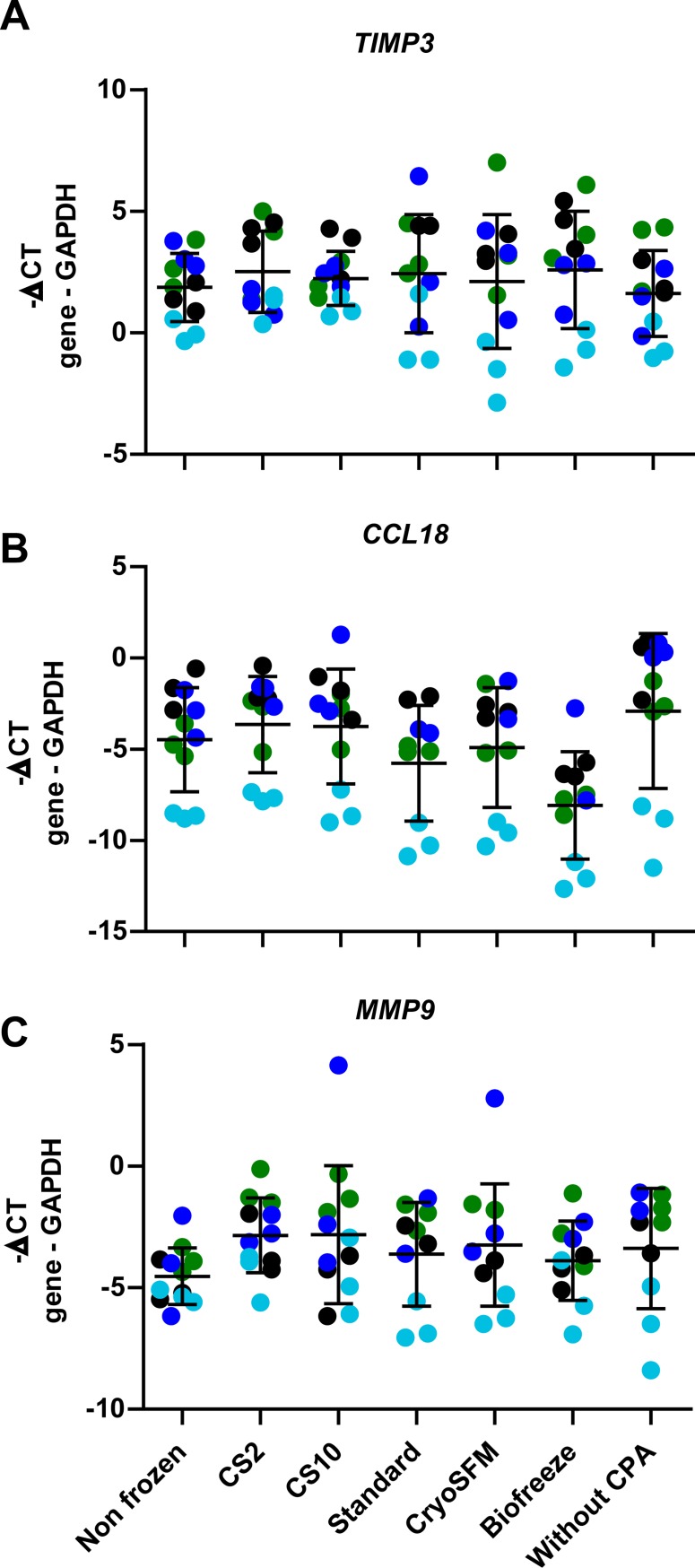
No effect of cryopreservation on disease characteristic gene expression. Gene expression of *TIMP3* (A), *CCL18* (B) and *MMP9* (C) was determined after thawing and 24 hours culture of synovial biopsies of four patients. Per patient 3 biopsies were included in the analysis. Patient 6: green dots, patient 7: black dots, patient 8: dark blue dots, patient 4: light blue dots. Displayed is the mean +/- SD. Statistical analysis was performed by one-way ANOVA, comparing the non-frozen control to the cryopreserved conditions.

**Fig 6:**
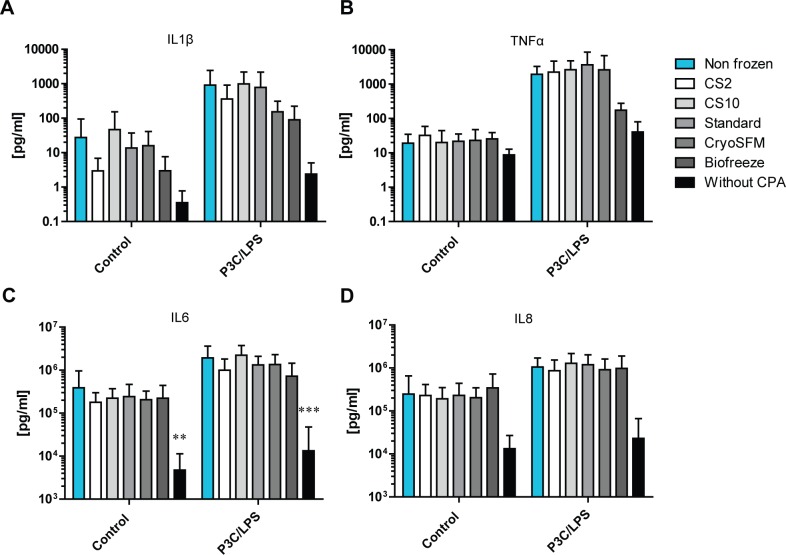
Cryopreserved synovial tissue is responsive to inflammatory stimuli. The secretion of proinflammatory cytokines IL1β (A), TNFα (B), IL6 (C) and IL8 (D) was determined before and after 24 hour stimulation with P3C/LPS. Displayed is the mean +/- SD. Statistical analysis was performed by Students t-test, comparing the unstimulated control to the stimulated conditions. ** p<0.01; *** p<0.001.

For statistical analysis GraphPad Prism version 5.03 was used. To determine significant differences between groups ANOVA was performed in combination with the Tukey test (Figs 1–5). To determine significant differences between non-stimulated and stimulated tissue for cytokine expression, the Student t-test was applied (Fig 6). Results are depicted as means +/- SD, and p values below 0.05 were regarded as significant.

## Results

### Effect of cryopreservation on tissue morphology and cell composition

We first determined the effects of cryopreservation on tissue morphology and histological integrity by comparing freezing with five different cryopreservation media and freezing without cryoprotectant to non-frozen biopsies. The histological tissue integrity was assessed for structural integrity of the different structures within the tissue and cell death using an arbitrary scoring system. A significantly lower histological score was found for the CS2, Biofreeze and without CPA condition compared to the non-frozen condition (Fig 1A–1F). When studying the individual patients highest differences were observed in patient 1. A relatively high cellularity and influx of inflammatory cells were present in the tissue of patient 1 (Fig 1B), which were not observed in the tissue of the second patient (Fig 1C), indicating that infiltrating cells might be more vulnerable to cryopreservation-induced damage. This was in line with the observation that the lowest histological scores were obtained for the parameter ‘morphology of infiltrating cells’ using the CS2 (patient 1: 0.4, p2: no infiltrating cells present, p3: 0.5) and Biofreeze medium (patient 1: 0.56, p2: no infiltrating cells present, p3: 0).

### Effect of cryopreservation on tissue viability

The metabolic activity reflects the global cellular viability of the tissue. The effect of cryopreservation on the metabolic activity of human OA synovial tissue from three patients was measured using the ATP and XTT metabolic assays (Fig 2). Both assays showed similar results: all conditions displayed a lower, though not significant decrease in metabolic activity compared to the non-frozen control and the condition cryopreserved without CPA solution showed a significant decrease in both assays.

### Effect of cryopreservation on RNA integrity

Cell death leads to rapid RNA degradation, so the RNA quality (RIN values) is a direct measure of cryodamage and low RIN values impair reliable gene expression analysis by microarray analysis. This analysis was performed to check whether the cryopreserved synovium is suitable for gene expression analysis by microarray studies, requiring minimal RIN values of 7. As depicted in Fig 3, high RIN values, well above 7, could be obtained for the non-frozen and cryopreserved tissues. No significant differences could be detected between the non-frozen condition and the cryopreserved conditions. However, not every tissue biopsy yielded enough RNA (> 5 ng/ul) to successfully determine the RIN. This was the case in the biopsies that were cryopreserved without protecting CPA solution. This might indicate that the amount of isolated RNA gives an indication of the number of intact cells in the tissue at the time of isolation. As depicted in Fig 3 the non-frozen, CS10 and CryoSFM conditions have a higher number of biopsies with an appropriate yield than the CS2, Standard and Biofreeze conditions. Furthermore, the average RIN value of the CryoSFM condition was the highest, although not significant and displayed the smallest standard deviation of all conditions tested (Fig 3). These results indicate that cryopreserved tissue can be used for gene expression analysis by qPCR. However, for microarray analysis several 3 mm biopsies might need to be pooled to obtain a sufficient amount of RNA.

### Effect of cryopreservation on stress gene expression

We compared the expression of 2 heat-shock proteins and 4 apoptosis-related genes in response to cryopreservation and CPA solutions. Compared to the non frozen control no differences could be detected in the cryopreserved conditions (Fig 4A–4F). Higher levels of stress gene expression were found in the negative control condition where the tissue was cryopreserved without protecting CPA solution). When studying the individual patients no other significant differences could be observed than already described above, except for the *MCL1* gene in patient 6. Here, a significant upregulation was identified for the CS2 and Biofreeze condition (Fig 4F). These results show that cryopreservation of OA synovial tissue does not induce upregulation of the selected stress genes indicating the absence of a stress response in the OA synovial tissue 24 hours after thawing.

### Effect of cryopreservation on disease characteristic gene expression

Analyzing tissue morphology, metabolic assays and stress gene expression does not reflect the interaction between cell types, typical for proper tissue functioning. Therefore, we have functionally analyzed the tissue by determining the expression of disease-characteristic genes.

Disease-characteristic genes were selected via microarray analysis performed on synovial samples from 10 OA patients and 7 control probands. *TIMP3*, *CCL18* and *MMP9* were highly expressed in all patients and showed a fold increase of 14.1, 9.11 and 7.56 respectively compared to the control samples, indicating that these genes are characteristic for the OA disease phenotype (data not shown). We analyzed in four patients whether the expression of these three genes would be influenced by cryopreservation. Cryopreservation did not influence the expression of these three genes as no significant changes could be observed compared to the non frozen control (Fig 5A–5C). When patients were analyzed separately significant differences could be observed (Table 1). The Biofreeze CPA solution showed the most changes in gene expression. Furthermore, most changes in gene expression were observed in biopsies from patient 7 while none were identified in patient 8, indicating differences in tissue sensitivity to cryopreservation between patients (Table 1).

**Table 1:** Analysis of cryopreservation-induced changes in disease- characteristic gene expression compared to non-frozen tissue in individual OA patients.

Symbol	Gene	P6	P7	P8	P4
***TIMP3***	TIMP metallopeptidase inhibitor 3	unchanged	• CS2 ↑ ** • Standard ↑ * • Biofreeze ↑ **	unchanged	unchanged
***CCL18***	chemokine (C-C motif) ligand 18	unchanged	• Biofreeze↓ **	unchanged	• Biofreeze ↓ **
***MMP9***	matrix metallopeptidase 9	• CS2 ↑ ** • CS10 ↑ *	unchanged	unchanged	unchanged

Expression of TIMP3, CCL18 and MMP9 was determined in four patients (P6-P8, P4, Fig 5). Unchanged refers to no changes in gene expression compared to the non frozen condition. Where a CPA solution is mentioned an arrow indicates whether the expression of the gene was upregulated or down regulated compared to non frozen tissue. Statistical analysis was performed by one-way ANOVA, comparing the non frozen control to the cryopreserved conditions.

*p<0.05

**p<0.01.

### Effect of cryopreservation on cytokine secretion

To analyze whether the inflammatory pathways, involved in the pathology of part of the OA patients, were affected by cryopreservation, we studied the spontaneous release of TNFα, IL1β, IL6 and IL8 into the culture medium (Fig 6A–6D). For IL6, a significantly lower cytokine secretion was only observed compared to the non-frozen control in the condition without CPA (Fig 6C). Although, compared to the non frozen control, clear decreases were observed for the other cytokines in the condition without CPA, these were not significant. However, no changes were observed in all conditions cryopreserved with a CPA solution compared to the non frozen control condition. When studying the individual patients (not shown) significant differences were detected for patient 7 for IL6 secretion which showed lower levels in the CS2 (228 ± 115 ng/ml), CS10 (378 ± 53 ng/ml) and CryoSFM (312 ± 83 ng/ml) condition compared to the non frozen control (1182 ± 626 ng/ml). For patient 4 TNF secretion was higher in the Biofreeze condition compared to the non frozen control (38 ± 6 pg/ml vs 18 ± 8 pg/ml).

The intrinsic capacity of synovial tissue to respond to TLR-ligands P3C and LPS by cytokine release was also tested after cryopreservation. Again, only for IL6 a significantly lower cytokine secretion was observed compared to the non frozen control in the condition without CPA while not for the other conditions cryopreserved with a CPA solution, nor for IL1β, TNFα and IL8 in the condition cryopreserved without CPA (Fig 6). Although a clear decrease compared to the non-frozen control was observed in the secretion of these cytokines as well. At the individual patient level the IL8 secretion was significantly decreased in patient 7 (70 ± 67 ng/ml vs 1619 ± 49 ng/ml) and 8 (8 ± 2 ng/ml vs 1358 ± 639 ng/ml) in the condition without CPA compared to the non frozen control. Furthermore, compared to the non frozen control, the IL6 secretion was decreased in the Biofreeze condition in patient 4 (536 ± 396 ng/ml vs 2430 ± 1062 ng/ml).

For every condition a clear increase in cytokine secretion was observed after stimulation, indicating the tissue is able to respond to inflammatory stimuli. However, the increase in IL1β secretion was not significant for the condition in which the synovium was cryopreserved in the standard medium and without CPA (p-values for all conditions are shown in S2 Table). For IL6 the increase was not significant for the Biofreeze and without protection condition and for IL8 the increase was not significantly different for the CS2 medium and without protection condition. These results indicated that except for TNF secretion, the tissue was not capable of responding to inflammatory stimuli after being cryopreserved without any protecting CPA solution. After cryopreservation with the CS10 and CryoSFM medium the tissue showed upregulation of all four cytokines studied.

## Discussion

In this study we validated our slow freezing protocol for human OA synovial tissue by using 4 commercially available and one homemade CPA solution. We determined the viability of the tissue after 24 hours culture at the histological, metabolic, gene expression and protein secretion level. Significantly lower histological scores were detected for the CS2 and Biofreeze medium, indicating these media are suboptimal for maintaining tissue morphology. The other assays performed could not reveal significant differences between conditions. However, in the stress- and disease characteristic gene expression analyses, trends were observed pointing at negative changes in gene expression compared to the non frozen control for the Biofreeze and CS2 medium as well. With the CS10, Standard and CryoSFM CPA solution only minor differences were observed compared to the non-frozen condition, indicating we have successfully cryopreserved human OA synovial tissue using these CPA solutions. Taking into account that the CS10 and CryoSFM media are produced under GMP conditions, which guarantees standardized and validated quality of CPA solutions, less batch-to-batch variation is expected compared to home-made CPA solutions containing FCS and DMSO.

As described above, the CS2 and Biofreeze medium most often showed a divergence from the non frozen condition. Although the exact composition of the commercially obtained media was unknown to us, the DMSO content of the media was described. The CS10 and standard medium contain 10% DMSO, the CryoSFM medium 7.5%, the Biofreeze medium does not contain DMSO and the CS2 medium only 2%. Without knowing about other differences in composition our results suggest that a low DMSO concentration is not beneficial for the cryopreservation of human OA synovium. The fact that the only difference between the CS2 and CS10 medium is the DMSO concentration strengthens this suggestion.

Our results show a lack of response to LPS/P3C in the conditions cryopreserved without protecting CPA solution. The secretion of IL1β, IL6 and IL8 were not significantly elevated in this condition. However, TNFα was. It might be that freeze-thawing stress in the unprotected tissue causes release of membrane-bound TNFα, which leads to a higher protein concentration, while no new protein is secreted. There are links described between increased cellular stress and higher levels of activated TACE/ADAM17 leading to higher protein concentrations of TNFR1, released together with TNFα [22].

When comparing the results of the XTT and ATP assay, which both reflect the metabolic activity of the tissue, there was one major difference. The ATP levels in patient 4 are the lowest in all conditions compared to the other patients while in the XTT assay the metabolic activity of patient 4 is comparable or higher compared to the other two patients. It might be that the difference in outcome can be explained by a difference in assay mechanism: the XTT assay is based on the cleavage of the tetrazolium salt XTT, probably at the plasma membrane, to an orange formazan dye by the reducing properties of NADH produced by mitochondria [23]. The ATP assay is based on the conversion of Luciferin to the light emitting Oxyluciferin by Ultra-Glo Luciferase. In this reaction ATP is the missing factor and becomes available after cell lysis. Furthermore, it has been described that cellular stress due to inflammation [24] or hypotonic swelling in lung epithelial cells [25], leads to active ATP release. Hypotonic swelling might also occur during thawing, upon removal of the CPA solution. Active release of ATP might be involved in the lowered ATP levels in patient 4.

We evaluated the expression of several known stress genes: The heat shock protein family, of which HSPA1A and HSP27 are members, is known to be induced in response to stress. Although HSPA1A is also described to be downregulated by cryopreservation in bovine blastocysts [16]. Heat shock proteins bind to denatured proteins as chaperones assisting in refolding and prevention of aggregation [26]. In our analysis we could not detect significant differences between conditions analyzing these genes, except for the upregulation of HSPA1A and HSP27 upon cryopreservation in the condition in which the synovial tissue was cryopreserved without CPA. CASP3, BAX, CD95 and MCL1 are apoptosis associated proteins. CASP3, BAX and CD95 are pro-apoptotic proteins while MCL1 is anti-apoptotic. Programmed cell death is a mechanism known to be upregulated when experiencing stressful conditions, including cryopreservation [10]. CASP3, BAX and CD95 were described to be upregulated upon cryopreservation while MCL1 was described to be down-regulated [21]. Similar to the heat shock proteins, no significant differences were identified between the cryopreservation conditions except for the non-protected condition in which an upregulation of all four apoptosis related genes was found, including for the anti-apoptotic gene MCL1. An explanation for this could be that there are differences between cell types in expression of pro- and anti-apoptotic genes resulting in upregulation of pro-apoptotic genes in one cell type and anti-apoptotic genes in different cells, resulting in the observation of upregulation of both genes. All together, analyzing stress gene expression after cryopreservation could only be used to detect major stress responses and was not applicable to identify minor qualitative differences between cryopreservation solutions. In contrast to the stress-related genes, the expression of disease-specific genes TIMP3, CCL18 and MMP9 was not significantly affected by cryopreservation and the only differences were observed between samples of individual patients. TIMP3 is a metallopeptidase inhibitor, suggested to have a protective effect against degradation of articular cartilage *in vivo* [27]. CCL18 is a chemokine belonging to the CC chemokine family. CCL18 is mainly produced by antigen presenting cells, to attract among others T-and B-cells [28,29]. MMP9 is a metallopeptidase involved in OA cartilage degeneration [30]. Our results indicate that these genes can be suitable to detect subtle cryoprotectant or cryopreservation effects other than cryotoxicity at an individual.

In our approach we cultured the tissue for 24 hours after thawing and subsequently performed analysis to determine the viability. This time point was chosen as it has been suggested by the manufacturer of the BioLife CPA solutions. Furthermore, measuring cellular viability within the first hours after thawing likely results in an overestimation of the viability [31]. Additionally, the 24 hour time point is a common culture time point in functional studies, although some parameters might be best studied at a different time point. For instance, a peak in Caspase 3 expression was described 18 hours post thawing in normal human dermal fibroblasts. However, also at 24 hours post thawing, levels were still elevated compared to the control [32]. We have not included more time points in our analysis due to scarcity of material.

A validated cryopreservation protocol is of high importance in research. In the first place to obtain reproducible results and in the second place cryopreservation offers the possibility to pre-select tissue at certain characteristics to be used in a study. Furthermore, if the same cryopreservation protocol is used it offers the possibility to combine synovial material obtained at multiple locations. An important effect we foresee by improving the availability of human synovial tissue for research is that this could lead to a decrease in the number of experimental animals. The possibility to perform more complex and larger experiments (for example drug compound testing) with human material instead of mice will prevent generation of results that cannot be translated to the human situation. Related to this is the increasing interest in precision medicine [33–35], followed by an increasing demand for human tissue. Precision medicine includes the adjustment and development of therapies and drugs for specific patient subsets with a similar genetic background, leading to for instance a similar response to treatment. This will improve healthcare by applying the best therapy to each patient, additionally this approach will be more cost effective and prevent possible side effects in patients not benefitting from a therapy. The development of precision medicine and the existing research lines using human synovial tissue of both pharmaceutical companies and universities are dependent on the availability of high quality patient material. By performing mechanistic and intervention studies, the development of new treatment options for OA would likely benefit from the use of cryopreserved synovial tissue.

We showed that a slow freezing cryopreservation protocol using commercially available CPA solutions and a homemade CPA solution can protect human OA synovial tissue from cryodamage. We have validated a protocol to determine the viability of the tissue after cryopreservation, including an extensive analysis of the tissue functionality after thawing. Three CPA solutions were identified that performed equally well. A next step is to perform a reproducibility study, in which other labs will use our cryopreservation protocol to test if our results are reproducible. Furthermore, this study can be used as a template to investigate the possibility to cryopreserve the even more complex synovial tissue of rheumatoid arthritis patients and isolated primary cells acquired by synovial tissue digestion.

## Supporting Information

S1 Table List of qPCR primers. (DOCX)

S2 Table p-values of increase in cytokine secretion after stimulation. (DOCX)
