# Supplementary material for: Functional Tissue Analysis Reveals Successful Cryopreservation of Human Osteoarthritic Synovium
Source: PLoS One. 2016 Nov 21;11(11):e0167076. doi: 10.1371/journal.pone.0167076 (PMC5117761; doi:10.1371/journal.pone.0167076)
Supplement: S1 Table — (DOCX) [file pone.0167076.s001.docx]

**S1 table. List of qPCR primers**

| **Gene symbol** | **Forward sequence** | **Reverse sequence** |
| --- | --- | --- |
| GAPDH | ATCTTCTTTTGCGTCGCCAG | TTCCCCATGGTGTCTGAGC |
| HSPA1A | CAAGGCCAACAAGATCACC | CTCGATCTCCCCTGCTC |
| HSP27 | CTGGATGTCAACCACTTCG | AGATGTAGCCATGCTCGTC |
| CASP3 | AGCACCTGGTTATTATTCTTGG | TACTGTTTCAGCATGGCAC |
| BAX | CTTCAGGGTTTCATCCAGGA | TTACTGTCCAGTTCGTCCC |
| CD95 | CTCTTTAAGACTGTTCTTACGTCTG | CTTGGAGTTGATGTCAGTCAC |
| MCL1 | GCTAGTTAAACAAAGAGGCTGG | CTTCTAGGTCCTCTACATGGA |
| TIMP3 | TCTCCTTGTCCCTGCTTCATG | AGGCCCTTGACTACACTCTCATCT |
| CCL18 | GGTGTCATCCTCCTAACCA | GTCGCTGATGTATTTCTGGAC |
| MMP9 | CTTCCAGTACCGAGAGAAAGCCTAT | CAGGACGGGAGCCCTAGTC |
