# Supplementary material for: Functional Tissue Analysis Reveals Successful Cryopreservation of Human Osteoarthritic Synovium
Source: PLoS One. 2016 Nov 21;11(11):e0167076. doi: 10.1371/journal.pone.0167076 (PMC5117761; doi:10.1371/journal.pone.0167076)
Supplement: S2 Table — (DOCX) [file pone.0167076.s002.docx]

**S2 table. p-values of increase in cytokine secretion after stimulation**

| **Control vs P3C/LPS stimulation** | **Cytokines** | | | |
| --- | --- | --- | --- | --- |
|  | **IL1β** | **TNFα** | **IL6** | **IL8** |
| **Non frozen** | 0,0431 | < 0.0001 | 0,0041 | 0,0011 |
| **CS2** | 0,0292 | 0,0026 | 0,0021 | *0,2380* |
| **CS10** | 0,0053 | 0,0001 | <0,0001 | 0,0002 |
| **Standard** | *0,0506* | 0,0120 | 0,0003 | 0,0010 |
| **CryoSFM** | 0,0035 | 0,0256 | 0,0003 | 0,0021 |
| **Biofreeze** | 0,0249 | < 0,0001 | *0,0502* | 0,0391 |
| **Without CPA** | *0,1103* | 0,0155 | *0,3212* | *0,4334* |

Statistical analysis was performed by Students t-test, comparing the cytokine secretion of control (unstimulated) synovium to P3C/LPS stimulated synovium. Non significant p values are displayed in italics.
